# Desmosomal Cadherins Are Decreased in Explanted Arrhythmogenic Right Ventricular Dysplasia/Cardiomyopathy Patient Hearts

**DOI:** 10.1371/journal.pone.0075082

**Published:** 2013-09-23

**Authors:** Alexia Vite, Estelle Gandjbakhch, Catherine Prost, Veronique Fressart, Pierre Fouret, Nathalie Neyroud, Françoise Gary, Erwan Donal, Shaida Varnous, Guy Fontaine, Paul Fornes, Françoise Hidden-Lucet, Michel Komajda, Philippe Charron, Eric Villard

**Affiliations:** 1 UPMC, University Paris 06, Hôpital Pitié-Salpêtrière, Paris, France; 2 INSERM, UMR_S956, ICAN, Hôpital Pitié-Salpêtrière, Paris, France; 3 Institut de Cardiologie (AP-HP), Hôpital Pitié-Salpêtrière, Paris, France; 4 Laboratoire d’histologie et de thérapie génique, University Paris XIII, Bobigny, France; 5 Département de Pathologie, University Paris V, Hôpital Necker enfants malades, Paris, France; 6 Service de Biochimie Métabolique, Unité de Cardiogénétique, Hôpital Pitié-Salpêtrière, Paris, France; 7 Service d’Anatomie Pathologique, Hôpital Pitié-Salpêtrière, Paris, France; 8 Département de Cardiologie, Hôpital Pontchaillou, Rennes, France; 9 Département de Pathologie, Hôpital universitaire, Reims; UAE University, Faculty of Medicine & Health Sciences, United Arab Emirates

## Abstract

**Aims:**

Arrhythmogenic right ventricular Dysplasia/cardiomyopathy (ARVD/C) is an autosomal dominant inherited cardiomyopathy associated with ventricular arrhythmia, heart failure and sudden death. Genetic studies have demonstrated the central role of desmosomal proteins in this disease, where 50% of patients harbor a mutation in a desmosmal gene. However, clinical diagnosis of the disease remains difficult and molecular mechanisms appears heterogeneous and poorly understood. The aim of this study was to characterize the expression profile of desmosomal proteins in explanted ARVD/C heart samples, in order to identify common features of the disease.

**Methods and Results:**

We examined plakophilin-2, desmoglein-2, desmocollin-2, plakoglobin and β-catenin protein expression levels from seven independent ARVD/C heart samples compared to two ischemic, five dilated cardiomyopathy and one healthy heart sample as controls. Ventricular and septum sections were examined by immunoblot analysis of total heart protein extracts and by immunostaining.

Immunoblots indicated significant decreases in desmoglein-2 and desmocollin-2, independent of any known underlying mutations, whereas immune-histochemical analysis showed normal localization of all desmosomal proteins. Quantitative RT-PCR revealed normal *DSG2* and *DSC2* mRNA transcript levels, suggesting increased protein turn-over rather than transcriptional down regulation.

**Conclusion:**

Reduced cardiac desmoglein-2 and desmocollin-2 levels appear to be specifically associated with ARVD/C, independent of underlying mutations. These findings highlight a key role of desmosomal cadherins in the pathophysiology of ARVD/C. Whether these reductions could be considered as specific markers for ARVD/C requires replication analysis.

## Introduction

Arrhythmogenic right ventricular dysplasia/cardiomyopathy (ARVD/C) is a rare inherited cardiomyopathy characterized by progressive sub-epicardial fibro-fatty replacement of myocardial tissue, mostly in the right ventricle [1,2,3]. The typical clinical presentation associates right ventricular arrhythmias with right ventricular morphological (dilatation and/or wall motion abnormalities) and electrocardiographic abnormalities (epsilon wave, T wave inversion and parietal block in right precordial leads). Left ventricular involvement is not rare ranging from 10 to 28% [4,5]. In a small number of subjects, the worsening of the right or biventricular dysfunction can lead to end-stage heart failure needing heart transplantation [5]. Diagnosis is based on a composite score calculated from electrocardiographic and morphological recordings as well as the familial disease pattern, histology and genetic screening results [6]. However, ARVD/C is clinically heterogeneous, and despite carefully selected criteria, diagnosis remains difficult, especially in moderate or borderline forms of the disease. This clearly highlights the need for an improved understanding of the mechanisms leading to the disease and more selective diagnostic criteria [7].

ARVD/C usually presents with an autosomal dominant mode of inheritance and incomplete penetrance [8,9,10]. A major breakthrough in elucidating the molecular pathogenesis of ARVD/C resulted from the identification of causative heterozygous mutations in genes encoding the cardiac desmosomal proteins; plakophilin-2 (PKP2), desmoglein-2 (DSG2), desmocollin-2 (DSC2), plakoglobin (JUP) and desmoplakin (DSP) [11]. Desmosomal mutations were firstly described in Naxos disease and Carvajal syndrome due to mutations in *JUP* and *DSP*, associating ARVD/C with palmoplantar keratosis and woolly hair [12,13]. Overall, mutations have only been identified in 40-60% of ARVD/C patients and, due to incomplete penetrance, do not completely solve the clinical diagnostic challenge. Despite these significant advances in the genetics basis of ARVD/C, how the mutant proteins contribute to the disease physiopathology remains largely unexplained.

Desmosomes are multi-protein complexes expressed in epithelial and cardiac tissues. They ensure strong cell-to-cell adhesion via homophilic and heterophilic interactions between the desmosomal cadherins DSG2 and DSC2 [14,15]. Such interactions are reported to stabilize the cadherins at the membrane [16,17]. Cadherins also interact with partners belonging to the armadillo protein family, such as catenins and plakophilins, described as mediators of intracellular signaling [18]. A few studies have implicated plakoglobin and Wnt/beta-catenin signaling in ARVD/C-related adipogenesis and fibrogenesis, two characteristic histological features of the disease [19,20,21]. In addition, transgenic animal models, which at least partially recapitulate the disease phenotype, have also confirmed a possible role for plakoglobin signaling in ARVD/C [19,22].

More than 40% of the mutations found in ARVD/C are nonsense or frame-shift, indicating that haploinsufficiency is a likely mechanism leading to the disease. Moreover, ARVD/C heart samples reproducibly exhibit smaller desmosomes with increased intermembranous space on electron-micrograph, perhaps resulting from an overall reduction in the length and adhesive properties of the desmosomes [23] [21]. Additional support for this theory comes from recent studies frequently reporting reduced plakoglobin levels at the intercalated discs in ARVD/C cardiac biopsies [24,25]. Based on these observations, we hypothesized that decreased desmosomal protein levels at intercalated disks could be a common feature of ARVD/C. We performed systematic immunolabeling and immunoblotting to assess levels and localization of intercalated disk proteins (JUP, PKP2, DSG2, DSC2 and β-catenin) in a collection of seven heart samples from patients with clinically confirmed ARVD/C. We identified a marked and specific decrease in the expression of cardiac desmosomal cadherins.

## Materials and Methods

### 2.1 Ethics statement

All patients have signed a written informed consent form enabling the use of their tissue for this study. This project was given the approval number 35-06 by the CCPPRB (*Comité Consultatif de Protection des Personnes dans la Recherche Biomédicale*) on behalf of the PHRC (Programme Hospitalier de Recherche Clinique).

### 2.2 Tissue samples

Cardiac tissue samples were collected between 2006 and 2009 from patients with confirmed ARVD/C (referred to as: ARVD/C 1 to 7) according to validated diagnostic criteria [6,26], and who underwent cardiac transplant. The control samples were derived from the explanted hearts of two patients with ischemic- and five with idiopathic-dilated cardiomyopathy (DCM), and from one donor with no documented heart disease (normal control). All except one of the DCM cases used as controls displayed biventricular dilatation. The remaining case had ischemic DCM and did not present into right ventricular dilatation.

All tissues were immediately frozen in liquid nitrogen or isopentane at -80°C. Mutational screening of the five desmosomal genes *PKP2* (NM_004572.3), *DSG2* (NM_001943.3), *DSC2* (NM_024422.3), *DSP* (NM_004415.2) and *PG* (NM_021991.2) was performed in all ARVD/C patients as previously described [8]. Briefly, genomic DNA was extracted from blood cells of each patient and subjected to PCR amplification of each exon and intron- exon junctions from the five genes screened. Sequencing of PCR products was performed using BigDye dideoxy-terminator chemistry (PerkinElmer) on an ABI 3830 DNA sequencer (Applied Biosystems). Analysis of the chromatograms was performed with SeqScape (PE Applied Biosystems). The exons of the five genes were thoroughly analyzed even when a mutation was identified in a given gene. A control group of 300 healthy and unrelated subjects (600 alleles) with Caucasian origin were genotyped as controls to check that mutations were not frequent polymorphisms. Moreover, absence of the identified mutations was also ascertained in the ESV server (URL:http://evs.gs.washington.edu/EVS/).

### 2.3 Protein extraction and immunoblot analysis

Snap frozen tissues were homogenized in SDS-Urea buffer (1% SDS, 8 mM Urea, 10 mMTris pH7.5, 140 mMNaCl, 5 mM EDTA, 2 mM EGTA) supplemented with a cocktail of protease inhibitors (1:1000) (Sigma-Aldrich®, MO, USA). Proteins were loaded on 9 or 10% polyacrylamide gels and, following electrophoresis, were transferred to nitrocellulose membranes. They were then blocked for two hours in 5% non-fat milk in PBS with 0.1% Tween (PBS-T). Blots were incubated with primary antibodies overnight at 4°C, rinsed and incubated with secondary antibodies conjugated to the infra-red dyes 680LT or 800CW (LI-COR® Biosciences, NE, USA). Blots were imaged using the Odyssey® Imager (LI-COR® Biosciences). Protein signal intensities were quantified with the ImageJ freeware (version 1.41, NIH, Bethesda, MD, USA, http://rsb.info.nih.gov/ij/). For each measurement, the target protein density ratio was calculated using either α-actinin-2 (ACTN2) or cardiac myosin-binding protein C (cMyBP-C) as loading controls. Western blots were performed in duplicate for each heart compartment (ie. septum, left and right ventricles) and for each subject. Two to three independent protein extractions were performed to determine the average relative protein level per compartment and per subject.

### 2.4: Immunofluorescent labeling

Frozen heart samples were cut into 5 µm sections, and fixed with 4% paraformaldehyde for 10 min at room temperature (RT), followed by incubation in 100% methanol for 10 min at -20°C. Tissues were permeabilized with 0.2% Triton X-100 in phosphate buffered saline (PBS) for 5 minutes at RT, then blocked for 30 min with 5% bovine serum albumin and 0.01% Triton X-100 in PBS, and finally incubated with primary antibodies overnight at 4°C (Table S1). Samples were washed in PBS and labeled with Alexa Fluor® conjugated secondary antibodies (Fisher Scientific, MA, USA) for 1 hour at RT. Tissue sections were imaged with the Olympus® IX50 microscope (Olympus®, Tokyo, Japan), and 3D deconvolution performed using the Metamorph software (Roper Scientific®, NJ, USA), by two independent investigators, who were blinded to the sample origin.

### 2.5 Electron microscopy procedure

Right and left ventricle samples from the ARVD/C patient (ARVD/C 6) were fixed in 2% glutaraldehyde in 0.1M phosphate buffer, pH 7.4at 4° for 4 hours, then incubated in 0.6% glutaraldehyde in phosphate buffer at 4°. Samples were post fixed in 1% osmium tetroxyde in cacodylate buffer during 1 hour, and then colored with uranyl-acetate for 1 hour. Tissues were then dehydrated in a series of alcohol baths from 30% to 100% then in 100% propylene oxyde and embedded in

*2.5. RNA extraction* Epon 812 resin before electron microscopy imaging (HITACHI 120kV HT 7700). *and quantitative RT-PCR*

Total mRNA was isolated from snap frozen hearts with TRIzol (Life Technologies®, CA, USA) and concentrated with the RNeasy® minikit (Qiagen®, Hilden, Germany). cDNA was synthesized from 500 ng of total mRNA with oligo[dT]_12-18_ priming and the SuperScript™ III reverse transcriptase (Life Technologies^TM^). Quantitative real-time PCR (RT-PCR) was performed using the LightCycler® 480 System (Roche Diagnostics, IN, USA) and the Brilliant II SYBR® Green qPCR Master Mix (Agilent technologies, CA, USA), according to the manufacturer’s instructions. Specific primers were designed to amplify *DSG2, DSC2* and *ACTN2* cDNAs (*DSG2*-F: 5’ atgacggctaggaacaccac3’; *DSG2*-R: 5’ gggtcagtttgtggctgact3’; *DSC2-F*: 5’ ttggagcatcaaacaaaggtc3’; *DSC2-R*: 5’ atagttttgggccgtgtcag3’; *ACTN2*-F: 5’ cagaggaagaccttcactgc3’; *ACTN2*-R: 5’ caattttgtggaaccgcatt3’). All data were normalized to *ACTN2* mRNA level using the 2^-ΔΔCT^ method [27].

### 2.6 Statistics

Differences between the two groups (ARVD/C and controls) were analyzed using the non-parametric Mann-Whitney Rank Sum test. A *p* value < 0.05 was considered as statistically significant. Data are expressed as the mean value ± standard error (SEM).

## Results

### 3.1 Patient characteristics

Patient clinical and genetic details are presented in Table 1. All seven ARVD/C patients displayed severe disease with multifocal ventricular tachycardia and severe right ventricular or biventricular heart failure. They underwent cardiac transplantation at ages ranging from 14 to 74 years old. The presence of extensive fibro-fatty myocyte replacement, predominantly seen in the right ventricle, but also in the left ventricle, confirmed the ARVD/C diagnosis in all patients (Figures S1 and S2). Three of the patients (ARVD/C1, 2 and 6) carried heterozygous *DSG2* mutations as previously described (Table 1) [8]. No causative mutations were identified in the remaining four ARVD/C patients.

**Table 1:** Clinical and histopathological details of patients ARVD 1 to 7 included in this study.

**ARVD/CPatient**	**Desmosomal mutation**	**Demographics**			**Familial history**	**ECG**	**Arrhythmias**			**Morphology**				**Histology**			**Major/Minor criteria**
		**sex**	**age at diagnosis**	**Age at Ht Tx**			**Ventricular arrhythmias**	**Atrial arrhythmias**	**ICD**	**RV**	**RVEF**	**LV involment**	**LVEF**	**RV**	**LV**	**lymphocytes**	
1	***DSG2* (c.690+1 G>A**)	M	50	73	none	AV block, paced	Multifocal VT from RV** and LV	none	yes	major RV dilatation and hypokinesia**	<25%	yes	30%	Extensive fibro-fatty replacement **	Mild fibrosis	-	3/0
2	***DSG2* (p.Arg49His**)	M	13	14	Grandfather: severe ARVD leading to Ht Tx*	iRBBB, epsilon wave**	Multifocal VT from RV**	none	no	major RV dilatation and hypokinesia**	13%	yes	35%	Extensive sub-epicardial fibro-fatty replacement **	Large regions with sub-epicardial fibro-fatty replacement Extensive fibrosis	+	4/1
3	none	M	46	53	none	RBBB, TWI V1-V4*, epsilon wave**	Multifocal VT from RV**, FV	AF	yes	major RV dilatation, anterior akinesia severe hypokinesia**	<25%	none	55%	Extensive fibro-fatty replacement **	Mild sub-epicardial fibro-fatty replacement	+	4/1
4	none	F	21	30	none	RBBB, epsilon wave**, TWI V1-V4*	Multifocal VT from RV**	none	yes	major RV dilatation and hypokinesia**	27%	none	65%	Extensive fibro-fatty replacement **	Mild sub-epicardial fibro-fatty replacement	-	4/1
5	none	F	38	39	none	epsilon wave**, iRBBB,	11008 bimorphic VEc/24H*, polymorphic NSVT	AF	yes	major RV dilatation anterior akinesia and global hypokinesia**	28%	yes	45%	Extensive fibro-fatty replacement of RV anterior wall **	Large regions with fibrosis Mild fatty infiltration	+	3/1
6	***DSG2* (p.Arg46Trp**)	M	39	48	Brother : SD at 22*	iRBBB, epsilon wave**, TWI V1-V6**	Multifocal VT from RV**	None	yes	major RV dilatation and hypokinesia**	13%	yes	45%	Extensive fibro-fatty replacement **	Mild fibrosis	+	5/1
7	none	M	46	49	none	iRBBB, TWI V1 to V6**, epsilon wave**	Multifocal VT from RV**	None	yes	major RV dilatation and hypokinesia**	<25%	yes	26%	Extensive fibro-fatty replacement **	Large regions with sub-epicardial fibro-fatty replacement	+	5/0

HtTx: Heart Transplantation; AV: Atrioventricular block; RBBB: right bundle branch block; iRRRB: incomplete RBBB; TWI: T-wave inversion; VT: ventricular tachycardia; NSVT: nonsustained VT; Vec: ventricular ectopies; FV: ventricular fibrillation; RV: right ventricular; LV: left ventricular; EF: ejection fraction; SD: sudden death; AF: atrial fibrillation; ICD: implantable cardiac defibrillator; F: female; M: male; *: minor criteria; **: major criteria.

### 3.2 Immunoblot quantitation

An immunoblotting study was performed on left and right ventricle, and in septum heart samples to identify potential ARVD/C-specific cardiac protein expression patterns.

**Figure 1:**
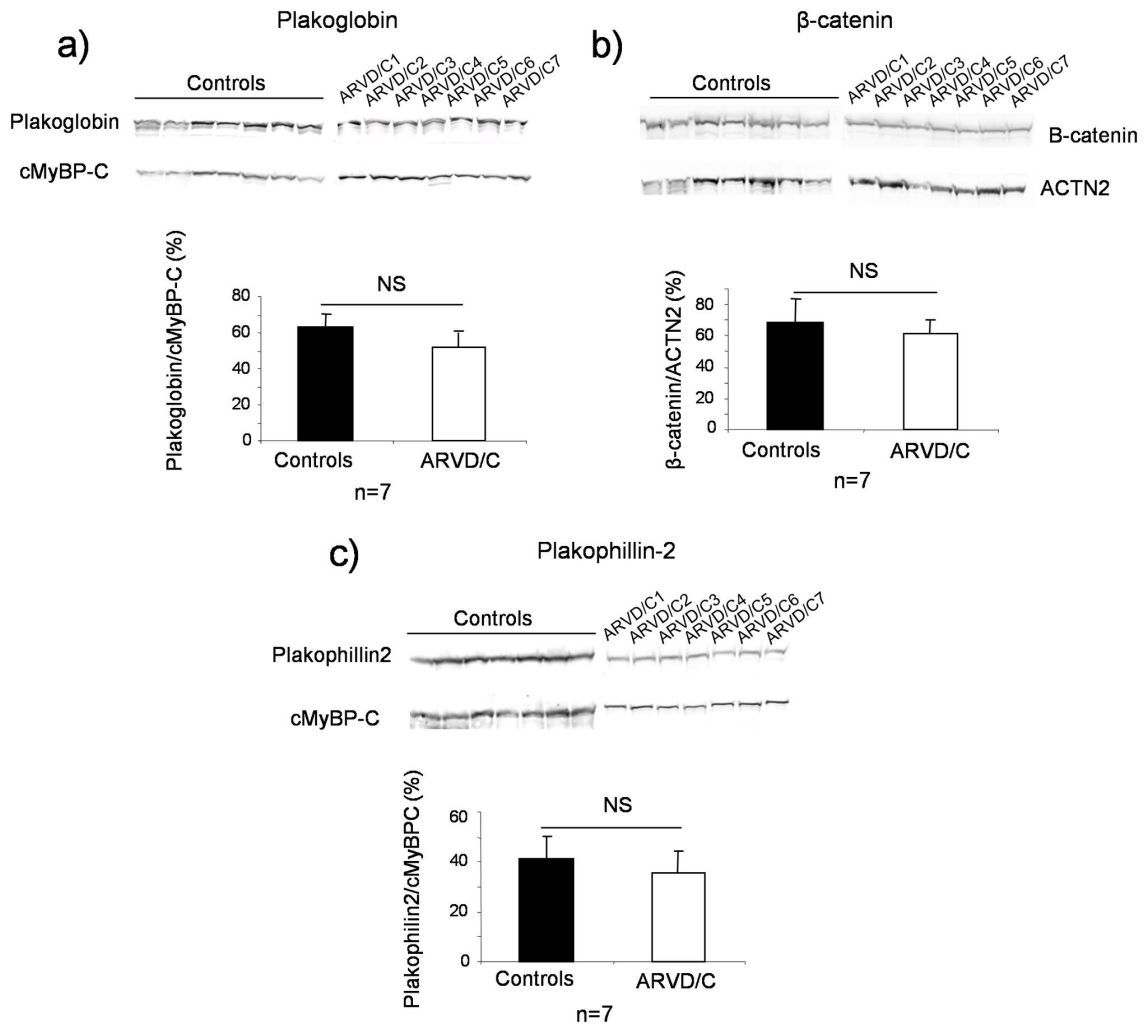
Quantitative immunoblotting of selected intercalated disk proteins; plakophilin-2, plakoglobin and β-catenin. Representative immunoblots obtained for (a) plakoglobin (left Ventricle), (b) β-catenin (septum) and (c) plakophilin-2 (septum). Bar graphs indicate the mean ± SEM following quantification of protein signals from all blots from the seven patient tissue samples and all compartments after normalization to the cardiac protein α-actinin-2 (ACTN2) or cardiac myosin-binding protein C (cMyBP-C) and to the control with the strongest signal level. NS for *p*>0.05, Mann Whitney test.

**Figure 2:**
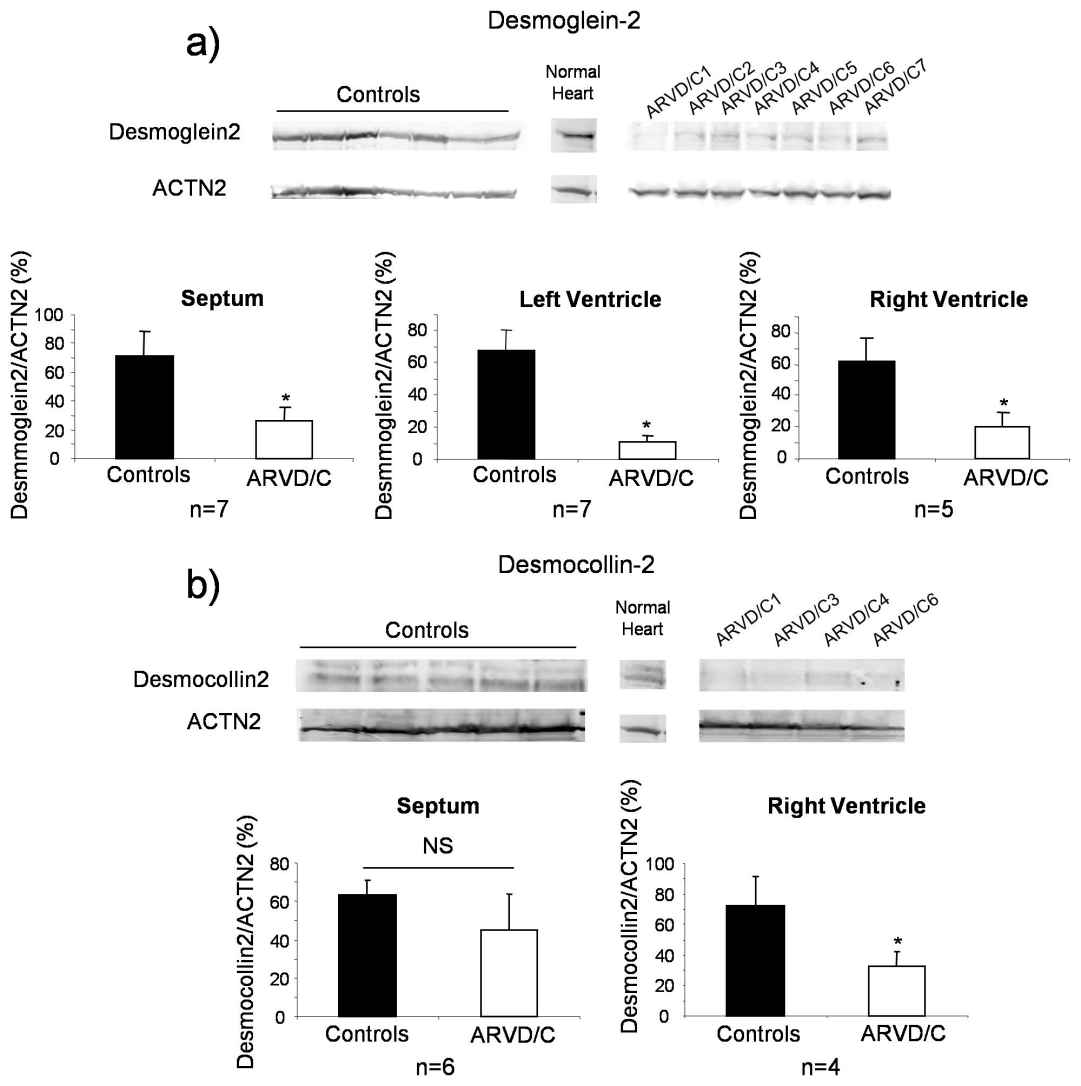
Quantitative immunoblotting of desmosomal cadherins desmoglein-2 and desmocollin-2. Representative images of western blots obtained for desmoglein-2 (a) and desmocollin-2 (b) in the right ventricle. Bar graphs indicate the mean expression ± SEM for each heart compartment from the seven patients, following normalization to the cardiac protein α-actinin-2 (ACTN2) and the control with the strongest signal level. *p<0.001, Mann Whitney Test.

As shown in Figure 1, total β-catenin, JUP and PKP2 protein levels were similar in ARVD/C and controls heart samples (all compartment together). There was neither any statistical difference in any compartments when assessed independently (data not shown). Conversely, the total DSG2 level was significantly decreased in all ARVD/C samples, compared to controls and normal heart, regardless of the disease-causing mutation (Figure 2a). Replicated densitometric measurement revealed an overall 74% decrease of desmoglein-2 signal in ARVD/C compared to controls samples, the ratio of DSG2 to α-actinin-2 (ACTN2) was 17.2 ± 6.6 in ARVD/C, 67.1 ± 9.8 in DCM samples (*p*<0.001, Mann Whitney Test) and 84.9 in the normal heart sample. Decreased DSG2 was observed for all patients (Figure S3) and in all cardiac compartments examined, but was more drastic in the left and right ventricles compared to the septum (Figure 2a).

**Figure 3:**
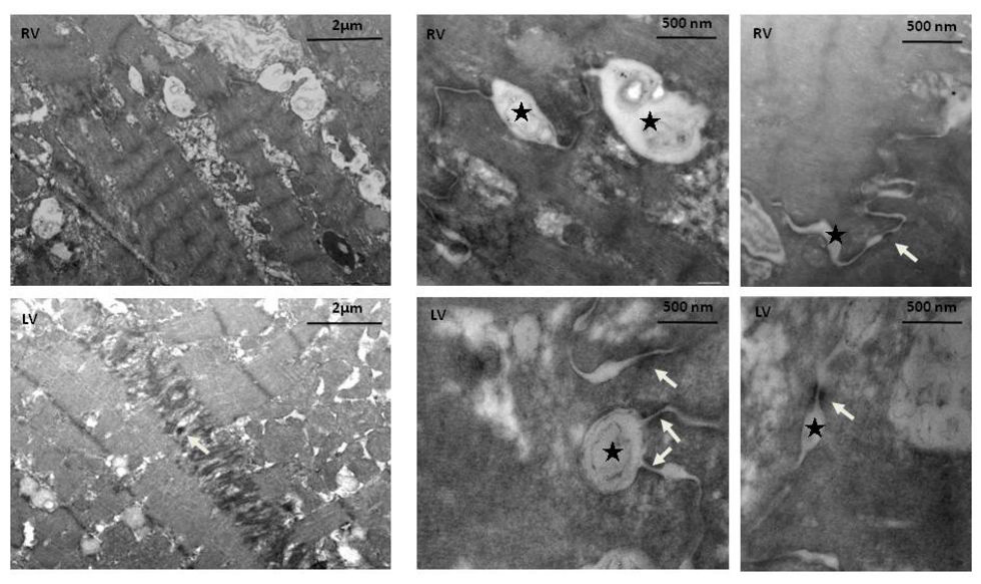
Electron microscopy of heart samples from ARVD/C 6. The electronic microscopy study of heart tissue from the p.Arg46Trp mutation-carrier (ARVD/C6) showed the presence of small desmosomes (white arrows) associated with abnormal inter-membrane vacuoles (black stars) in both ventricles (LV; left ventricle, RV; right ventricles).

Additionally, using electron microscopy on ARVD/C 6 patient, we have observed a clear reduction in desmosomal dense material at intercellular junctions; this was observed in both ventricles. Moreover we also observed opening inter-membrane vacuoles suggesting cellular disjunction (Figure 3).

Total DSC2 protein was also measured by immunoblot in the septum and right ventricle of only four ARVD/C patient samples due to the limited quantity of available tissue. The total DSC2 protein level was decreased (-43%) compared to controls. The ratio of DSC2 to ACTN2 was 38.7 ± 13.8 in ARVD/C, 68.1 ± 12.9 in controls samples (*p*<0.001, Mann Whitney Test) and 122.8 in the normal heart sample (Figure 2b). This decrease was statistically significant only in the right ventricle.

### 3.3 Intercalated disc protein Localization

**Figure 4:**
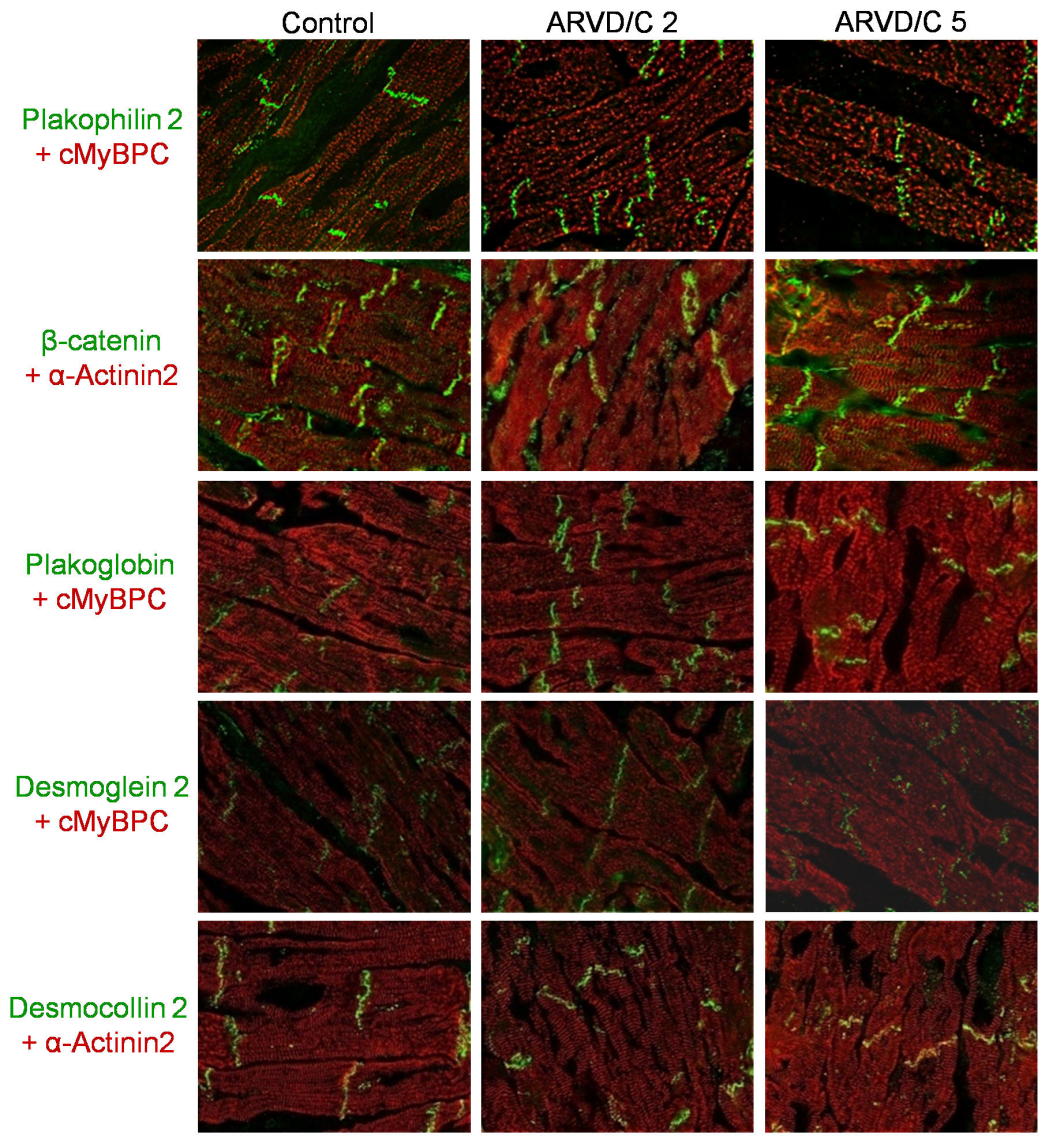
Representative immunolocalization of intercalated disc proteins in ARVD/C patient right ventricles. Representative pictures of plakophilin-2, β-catenin, plakoglobin, desmoglein-2 and desmocollin-2 labeling are shown (in green). Cardiac myosin-binding protein C (cMyBP-C) and α-actinin-2 (both red labeling) were used as cardiomyocyte specific markers. The figure shows heart tissue from two patients with ARVD/C compared to a representative (non ARVD/C) control.

To delve further into the characterization of the cadherin protein reduction, we performed immunohistochemical studies on the left and right ventricles (Figure 4). In addition to four desmosomal proteins found mutated in ARVD/C, (PKP2, DSG2, DSC2 and JUP), we also labeled β-catenin because of its possible functional implication in ARVD/C [28,29]. To control for tissue quality and localize cardiomyocytes, immunolabeling for ACTN2 or cardiac myosin-binding protein C (cMyBP-C) was also performed. All desmosomal proteins (PKP2, JUP, DSG2 and DSC2) and β-catenin showed normal localization at intercalated disks in both ARVD/C and control samples (Figure 4). Using the limit dilution of 1/300 for the antibody directed against DSG2, we observed a complete loss of immunolabeling for the 3 samples displaying the weakest amount of DSG2 on immunoblot (ARVD/C 1, 5 and 6, Figure S3), suggesting a correlation between the two measurement techniques (Figure S4).

### 3.4 *DSG2* and *DSC2* mRNA transcripts in ARVD/C.

**Figure 5:**
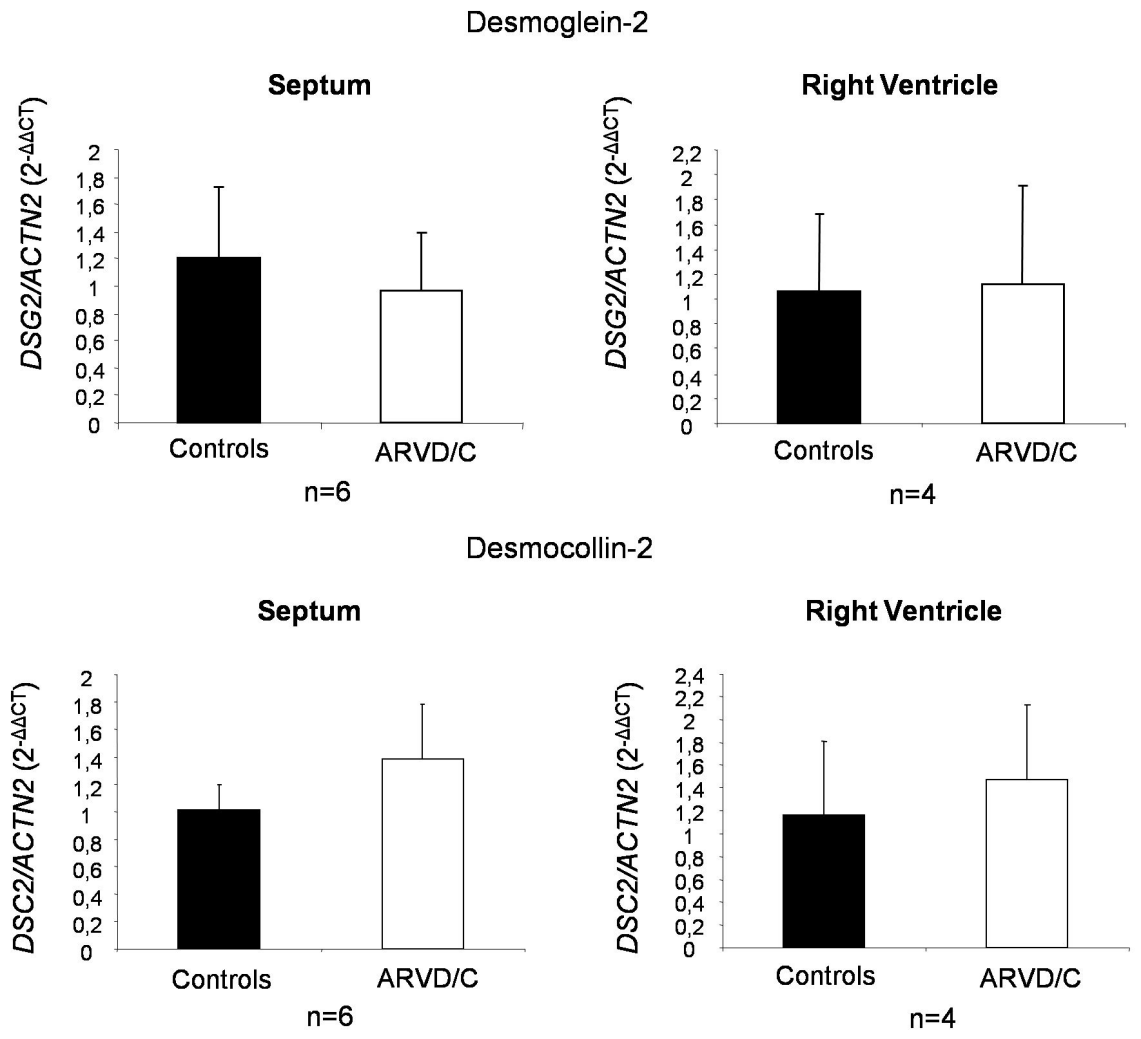
Quantitative RT-PCR analysis of *DSG2* and *DSC2* relative to *ACTN2* mRNA transcripts. Relative quantification was performed using RNA isolated from the septum or right ventricle of heart samples. Bar graphs show the mean ± SEM of six samples (2^-ΔΔCT^ method). *p*>0.05, Mann Whitney Test.

The decrease in desmosomal cadherins observed in ARVD/C samples could either be the consequence of transcriptional down-regulation or increased protein turnover. To answer this question, we studied *DSG2* and *DSC2* mRNA transcript level in four ARVD/C patient right ventricle samples (ARVD/C1 to 4) and in six septum samples (all patients except ARVD/C 3) by quantitative RT-PCR. *ACTN2* expression was used to standardize for cardiomyocyte specific mRNA input. As shown in Figure 5, there was no significant difference in *DSG2* and *DSC2* mRNA transcript level in ARVD/C compared to DCM control samples. This indicated that reduced levels of DSG2 and DSC2 in ARVD/C are not a consequence of mRNA down-regulation, but rather suggest an increased degradation or translation defect.

## Discussion

We characterized desmosomal protein expression in heart tissue samples from end-stage ARVD/C patients with or without mutations in desmosomal protein genes. We observed a significant decrease in DSG2 and DSC2 protein levels in all ARVD/C samples, regardless of the genetic status, but no mislocalization of desmosomal proteins, in comparison to the seven controls.

### Correlation between gene and protein expression

Mutations were not identified in the coding sequences of desmosomal genes (*PKP2*, *DSG2*, *DSC2*, *DSP* and *JUP*) in four ARVD/C cases, suggesting that the observed desmosomal cadherin decrease is an indicator of ARVD/C which is independent of the causative mutation. Nevertheless, it still remains unexplained why *DSG2* from transplanted ARVD/C subjects seems more frequently mutated in our patients (three of seven) compared to that which has been reported in previous ARVD/C screens (around 10% [8,30]), and to *PKP2*, the major gene in ARVD/C (around 30% [8]). It may be that *DSG2* mutations are associated with a more severe phenotype, thus requiring transplantation, as has already been reported by our team in a clinical analysis of 135 genotyped ARVD/C patients [8]. Confirming this observation with a larger study would be of great interest in defining phenotype-genotype correlations in ARVD/C.

### Specificity of desmosomal cadherin decrease in ARVD/C

DSG2 and DSC2 were the only desmosomal proteins shown to be significantly reduced by immunoblot in our study. This result suggests that desmosomal cadherin decrease is a specific feature of late-stage ARVD/C, rather than a general marker of cardiomyopathy-induced cardiac remodeling. None of the desmosomal proteins appeared altered by immunohistochemistry, suggesting no abnormal relocalization of them.

Although it was not our intention to quantify the signal on tissue immunostaining, we did not observe systematic decreased intensity of any specific desmosomal protein in all ARVD/C patients compared to controls. In order to increase our sensitivity to detect a decreased signal specific for DSG2 we applied a dilution protocol for the antibody directed against DSG2 as proposed by Asimaki et al. [24]. At maximum dilution only 3 out of 6 ARVD/C samples showed clear loss of fluorescent signal (Figure S4). Interestingly, these samples correlated to ARVD/C samples 1, 5 and 6 which display the most drastic decrease in DSG2 as measured by immunoblotting (Figure S3). Therefore this suggests a correlation between immunoblotting and immunolabeling that was only partial due to a threshold effect and insufficient sensitivity of our antibody. We also anticipated a reduced plakoglobin labeling, as described by Asimaki et al. [24]. However our immunolabeling protocol was slightly different and perhaps the primary antibody dilution we used may not have been adequately sensitive [24]. Therefore, the described decrease of plakoglobin at intercalated discs of ARVD/C cardiomyocytes, together with our immunoblot results, showing stable overall levels of plakoglobin protein, suggest a relocalization of the protein rather than a degradation, as was proposed by Asimaki et al. [24,25].

We also observed that among the desmosomal proteins tested, only cadherins were specifically decreased. This result is partly supported by another study performed on an isolated case with compound heterozygous mutations in *DSC2* and *DSG2*, showing decreased DSG2 expression with unchanged plakoglobin and PKP2 [31]. Compare to DSG2, the decrease in DSC2 was less significant and restricted to right ventricle. This is possibly correlated with increased severity of the phenotype in this compartment which is primarily altered in ARVD/C. Similar observation was made by Gehmlich et al. reporting a complete DSG2 extinction associated with a strong but incomplete decrease in DSC2 in a single explanted heart from a human ARVD/C case carrying a heterozygous DSG2 null mutation [32].

We studied samples with advanced forms of ARVD/C from patients carrying *DSG2* or other undetected mutations. It remains to be determined if the desmosomal cadherins decrease, particularly of DSG2, is also observed in patients with mutations in genes encoding desmosomal proteins other than cadherin, and if this reduction is discernible in earlier forms of ARVD/C.

### Physiopathological mechanisms of desmosomal cadherin decrease in ARVD/C

Desmosomal cadherin decrease is likely due to increased protein degradation or to translational defects, as the levels of *DSG2* and *DSC2* transcripts were similar to those measured in controls. Normal levels of cadherin mRNA in the right ventricle of ARVD/C patients has also been previously reported [33]. This is correlated with electronic microscopy studies on ARVD/C biopsies [23,30,34] which clearly indicate a progressive decrease in desmosomal length associated with disrupted intercellular junctions. In our study, desmosomal cadherins appear central to the pathophysiological mechanism of ARVD/C. However, the exact mechanism of the reduction remains unclear, and needs to be confirmed in larger studies. An attractive hypothesis could be that non-functional desmosomes (either due to mutant protein incorporation acting through a dominant negative effect, or to decreased expression of key structural components such as PKP2), are internalized with subsequent selective degradation of the cadherins, while other desmosomal components are recycled. Accordingly, via electron microscopy we observed such reduction in desmosomal materials in one of our studied explanted tissue (ARVD/C6). Such a mechanism is observed for desmoglein-3 in the life-threatening dermatosis pemphigus vulgaris, where the inhibition of cadherin adhesion (resulting from interaction of anti-cadherin auto-antibodies) leads to cadherin internalization and degradation, while plakoglobin steady state levels remain unaltered [16,35].

Altogether, our results suggest that a key aspect of ARVD/C pathophysiology could be increased degradation of cardiac cadherins, leading to desmosomal disorganization and disrupted cardiomyocyte junctions, and which may be a marker of ARVD/C. In order to gain insight into ARVD/C physiopathology, further studies are required to confirm this finding and identify the downstream pathways affected by desmosomal cadherin loss.

### Study limitations

Decreased cadherin expression is observed in all end-stage ARVD/C patient heart samples and not in the normal heart or dilated cardiomyopathy samples. We had access to seven heart tissue samples from ARVD/C patients and only one normal heart, low numbers which weaken our conclusions. Additionally, mutations in desmosomal genes have been recently reported in 5% of DCM cases [36], and we cannot exclude that the explanted DCM heart samples originated from donors carrying desmosomal gene mutations. However, given the low prevalence, the probability that one DCM donor carried a desmosomal gene mutation is small.

Finally, we cannot exclude that patients with no identified mutations, may carry undetected genetic abnormalities such as large deletions in *DSG2* and *DSC2*, or mutations in unknown genes which could affect *DSC2* and *DSG2* expression.

## Supporting Information

Figure S1 **Representative histology images of right ventricles from ARVD/C1-4.** Hematoxylin and eosin-stained section of the right ventricular myocardium showing mostly fatty replacement in all ARVD/C patients. Two magnifications are represented (2X and 10X).
(TIF)

Figure S2 **Representative histology images of right ventricles from ARVD/C5-7.** Hematoxylin and eosin-stained section of the right ventricular myocardium showing mostly fatty replacement in all ARVD/C patients. Two magnifications are represented (2X and 10X).
(TIF)

Figure S3 **Quantitative immunoblotting of desmoglein-2 in all ARVD/C and control patients.** Representative images of western blots obtained for desmoglein-2 in the right, left ventricle and septum for each patient. Bar graphs indicate the mean ± SEM following normalization to the cardiac protein α-actinin-2 (ACTN2).
(TIF)

Figure S4 **Representative images of immunofluorescent staining in ARVD/C and control patients after maximum dilution of anti-desmoglein-2 antibody.** Immunofluorescent staining of desmoglein-2 (DSG2) was performed in all ARVD/C and control samples as described in the material and methods section of the manuscript. We used the maximum dilution (before total extinction of the signal in controls) of anti-DSG2 antibody (1/300). The labeling of three samples, ARVD/C1, 5 and 6, appeared reduced compare to the others (ARVD/C2, 3, 4 and controls 1 to 6). These three samples match the samples for which the immunoblot based quantification of DSG2 appeared lower (Figure S3).
(TIF)

Table S1 **Primary antibodies used in study.**
(DOC)
