# Supplementary figures and images for: Desmosomal Cadherins Are Decreased in Explanted Arrhythmogenic Right Ventricular Dysplasia/Cardiomyopathy Patient Hearts
Source: PLoS One. 2013 Sep 23;8(9):e75082. doi: 10.1371/journal.pone.0075082 (PMC3781033; doi:10.1371/journal.pone.0075082)

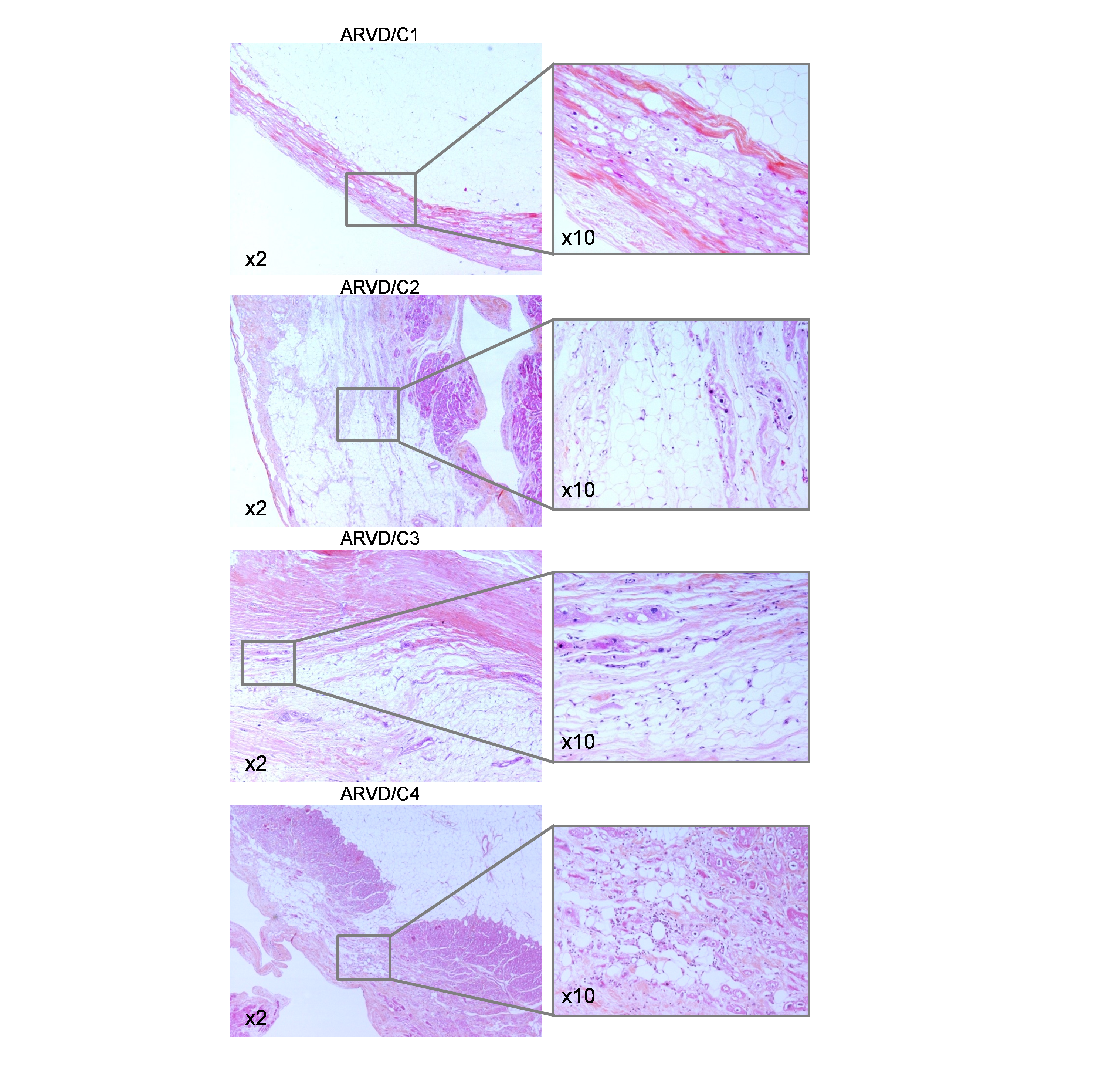

Supplement: Figure S1 — Representative histology images of right ventricles from ARVD/C1-4. Hematoxylin and eosin-stained section of the right ventricular myocardium showing mostly fatty replacement in all ARVD/C patients. Two magnifications are represented (2X and 10X). (TIF) [file pone.0075082.s001.tif]

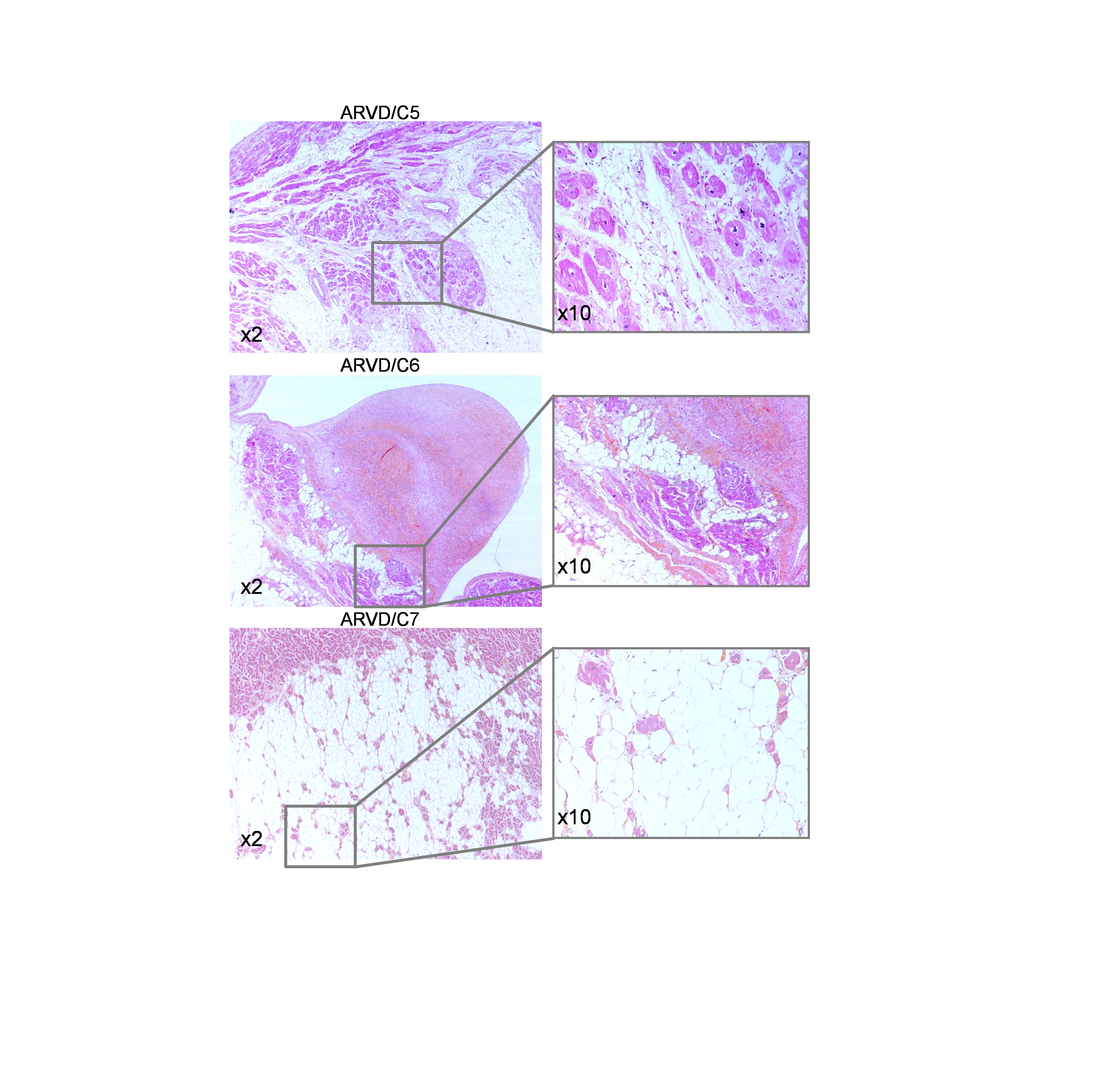

Supplement: Figure S2 — Representative histology images of right ventricles from ARVD/C5-7. Hematoxylin and eosin-stained section of the right ventricular myocardium showing mostly fatty replacement in all ARVD/C patients. Two magnifications are represented (2X and 10X). (TIF) [file pone.0075082.s002.tif]

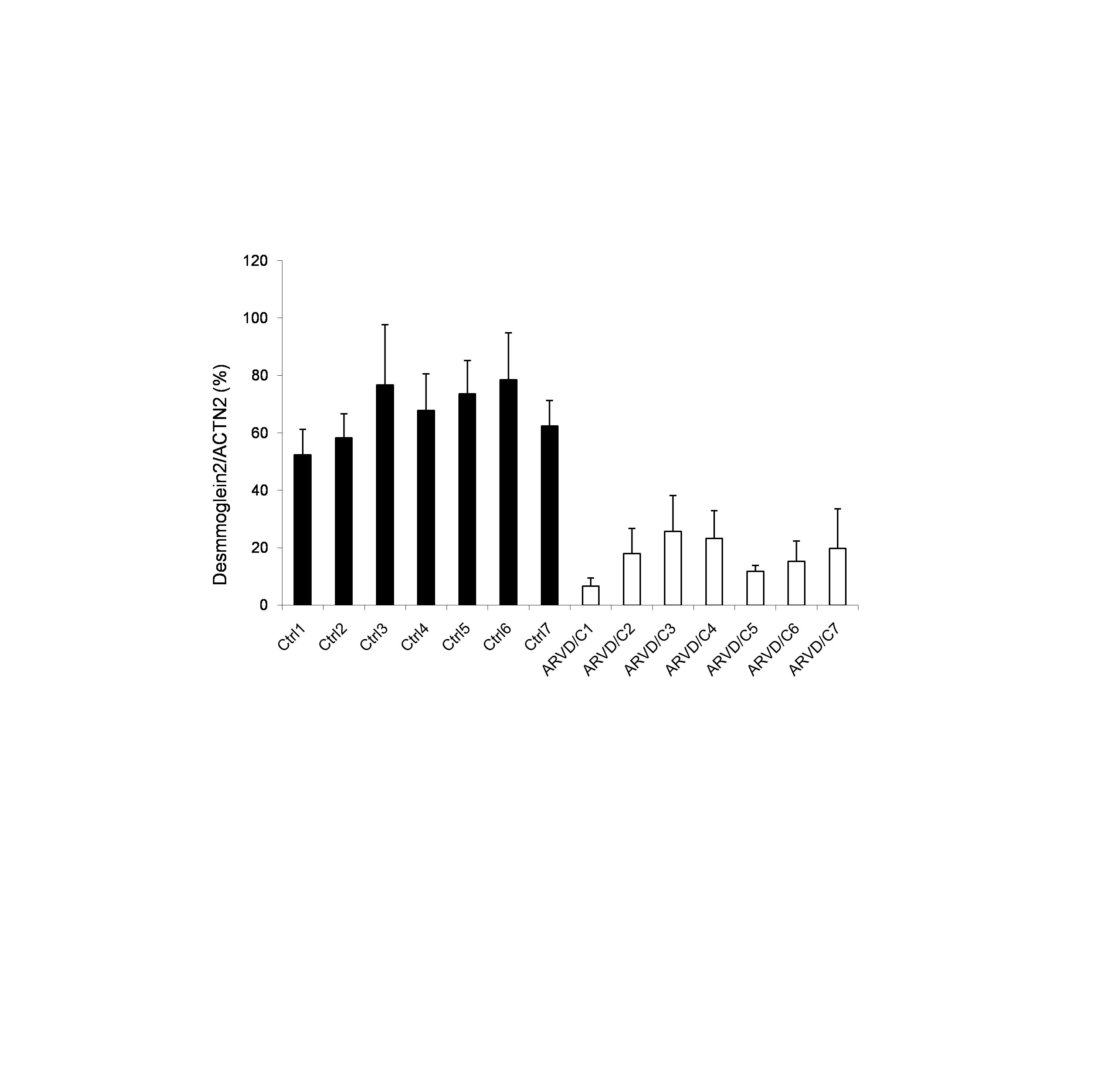

Supplement: Figure S3 — Quantitative immunoblotting of desmoglein-2 in all ARVD/C and control patients. Representative images of western blots obtained for desmoglein-2 in the right, left ventricle and septum for each patient. Bar graphs indicate the mean ± SEM following normalization to the cardiac protein α-actinin-2 (ACTN2). (TIF) [file pone.0075082.s003.tif]

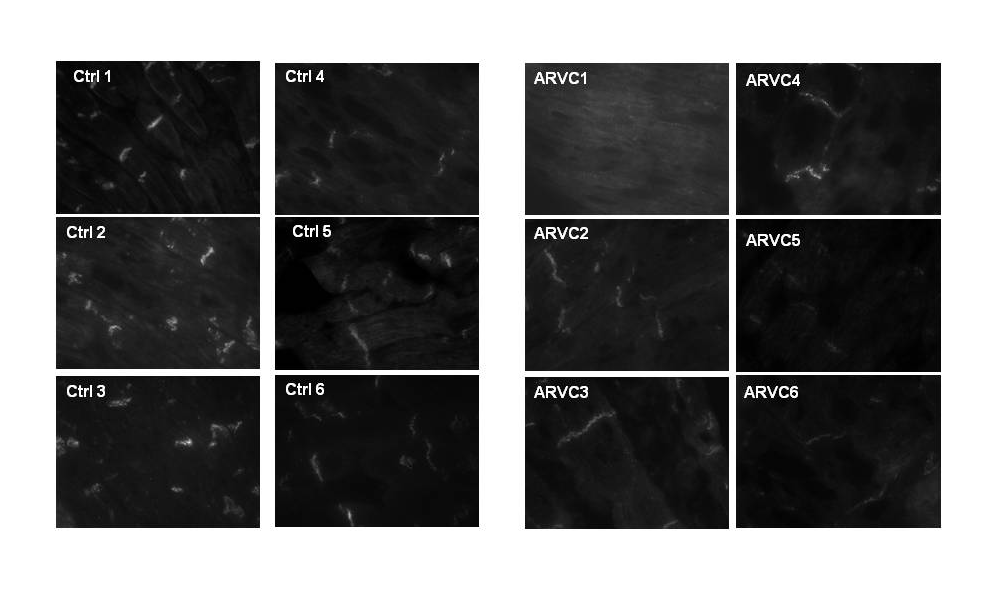

Supplement: Figure S4 — Representative images of immunofluorescent staining in ARVD/C and control patients after maximum dilution of anti-desmoglein-2 antibody. Immunofluorescent staining of desmoglein-2 (DSG2) was performed in all ARVD/C and control samples as described in the material and methods section of the manuscript. We used the maximum dilution (before total extinction of the signal in controls) of anti-DSG2 antibody (1/300). The labeling of three samples, ARVD/C1, 5 and 6, appeared reduced compare to the others (ARVD/C2, 3, 4 and controls 1 to 6). These three samples match the samples for which the immunoblot based quantification of DSG2 appeared lower (Figure S3). (TIF) [file pone.0075082.s004.tif]
