# Supplementary material for: Desmosomal Cadherins Are Decreased in Explanted Arrhythmogenic Right Ventricular Dysplasia/Cardiomyopathy Patient Hearts
Source: PLoS One. 2013 Sep 23;8(9):e75082. doi: 10.1371/journal.pone.0075082 (PMC3781033; doi:10.1371/journal.pone.0075082)
Supplement: Table S1 — Primary antibodies used in study. (DOC) [file pone.0075082.s005.doc]

| **Antigen** | **Antibody/Clone** | **Source** | **Dilution (Immunofluorescence)** | **Dilution (Immunoblot)** |
| --- | --- | --- | --- | --- |
| Desmoglein-2 | mAb/6D8 | Hycult biotechnology | 1:200 | 1:500 |
| Desmocollin-2 | pAb/ab95967 | Abcam | 1:100 | 1:200 |
| Plakoglobin | mAb/15F11 | Sigma | 1:200 | 1:1000 |
| Plakophilin-2 | mAb/PKP2a+PKP2b | Progen | 1:4 | 1:20 |
| β-catenin | pAb/ab6302 | Abcam | 1:200 | 1:500 |
| α-actinin 2 | mAb/ACTN2 | Sigma | 1:200 | 1:1000 |
| cMyBP-C | pAb | Custom* | 1:200 | 1:2000 |

Table S1: Primary antibodies used in study

mAb: monoclonal antibody; pAb: polyclonal antibody; *kindly donated by C. Witt & W. Linke (Heidelberg Germany)
